# The Role of Anti-U1 RNP Antibody in Connective Tissue Disease-Associated Pulmonary Arterial Hypertension: A Systematic Review and Meta-Analysis

**DOI:** 10.3390/jcm12010013

**Published:** 2022-12-20

**Authors:** Weizhen Xiang, Rongrong Dong, Meiqi Li, Baocheng Liu, Zhenzhen Ma, Qingrui Yang

**Affiliations:** 1 Department of Rheumatology and Immunology, Shandong Provincial Hospital, Shandong University, Jinan 250021, China; 2 Department of Rheumatology and Immunology, Shandong Provincial Hospital Affiliated to Shandong First Medical University, Jinan 250021, China

**Keywords:** connective tissue disease, pulmonary arterial hypertension, anti-U1 ribonucleoprotein antibody, meta-analysis

## Abstract

Connective tissue disease (CTD) patients may suffer from pulmonary arterial hypertension (PAH), a serious complication, and anti-U1 ribonucleoprotein (RNP) antibodies can be used as a potential indicator for the development and prognosis of CTD-associated PAH (CTD-PAH). However, there are still some controversies; thus, a systematic review and meta-analysis were performed. We searched PubMed, Embase, Cochrane Library, and Scopus for eligible studies and assessed their quality using Newcastle–Ottawa scales or Agency for Healthcare Research and Quality indicators according to the type of research. Odds ratio (OR) was adopted as a measure of effect in risk factor analysis, and hazard ratio (HR) was adopted for prognostic factor analysis. Publication bias was evaluated using the Egger’s test. Thirteen studies were finally included. Anti-U1 RNP antibody was proved as a risk factor for PAH among CTD patients (OR = 5.30, 95%CI 2.96–9.48, *p* < 0.05) and a protective factor against mortality among CTD-PAH patients (HR = 0.55, 95%CI 0.36–0.83, *p* < 0.05). CTD patients with positive anti-U1 RNP antibodies are at high risk for PAH, so routine screening examinations, including echocardiography, are recommended. Additionally, anti-U1 RNP positivity has been linked to decreased mortality in patients with CTD-PAH.

## 1. Introduction

Pulmonary arterial hypertension (PAH) is a progressive disease characterized by increased pulmonary arterial pressure and pulmonary vascular resistance resulting from loss and obstructive remodeling of the pulmonary vascular bed. According to the latest 2022 guideline of the European Society of Cardiology and the European Respiratory Society (ESC/ERS), PAH is defined by a mean pulmonary arterial pressure (mPAP) above 20 mmHg at rest, 15 mmHg or lower pulmonary artery wedge pressure (PAWP), and pulmonary vascular resistance (PVR) greater than 2 WU [1]. However, the majority of existing research take the previous 2015 ESC/ERS definition as diagnostic criteria, in which PAH is defined as at or above 25 mmHg of mPAP, 15 mmHg or less of PAWP, and greater than 3 WU PVR [2].

PAH is a common and serious complication of connective tissue diseases (CTDs), mainly occurring in patients with systemic sclerosis (SSc), systemic lupus erythematosus (SLE), primary Sjogren’s syndrome (pSS), and mixed connective tissue disease (MCTD) [3]. In previous studies, SSc accounted for up to 74% of patients with connective tissue disease-associated pulmonary arterial hypertension (CTD-PAH) in Europeans and Americans [4], while SLE had the highest prevalence among Asians (29% to 49%) [5,6]. With a 3-year survival rate of 52%, SSc-PAH has a poorer prognosis than other types of CTD-PAH [7]. Even though CTD-PAH is associated with high morbidity and mortality, early diagnosis is tricky because of non-specific clinical symptoms such as fatigue, dyspnea, and syncope; therefore, the diagnosis is frequently delayed and severe right ventricular dysfunction has usually already formed at the time it is identified [8]. Therefore, it is necessary to identify potential indicators for diagnosis and prognosis of the disease.

Anti-U1 ribonucleoprotein (RNP) antibody was first detected in MCTD, and later found in other CTDs including SSc and SLE. Numerous studies have shown an association between anti-U1 RNP antibodies and pulmonary damage [9,10], but also better survival [11,12]. There is, however, conflicting evidence in other research as to the role of anti-U1 RNP antibodies in CTD-PAH [13,14]. In this study, a systemic review and meta-analysis were conducted to determine whether anti-U1 RNP antibody is a risk factor and whether it is a prognostic factor of survival for CTD-PAH.

## 2. Materials and Methods

This meta-analysis has been registered on the PROSPERO registry (CRD42022324072), and the results were reported in accordance with the preferred reporting items for systematic review and meta-analysis (PRISMA) protocols [15].

### 2.1. Information Source and Search Strategy

Databases including PubMed, Embase, Cochrane Library, and Scopus were searched for eligible studies; the last search was conducted on September 5, 2022. The search strategy of PubMed is presented below as an example, and the detailed strategy can be found in Table S1.

1. (anti-U1 RNP antibody) OR (anti-nRNP antibody) OR (anti-RNP antibody) OR (anti-ribonucleoprotein antibody)
2. (pulmonary arterial hypertension) OR (pulmonary hypertension) OR (PAH)
3. 1 and 2

### 2.2. Inclusion and Exclusion Criteria

Eligible studies met the following conditions: (a) contained CTD patients with and without PAH for analysis of risk factors, and/or survivors and non-survivors with CTD-PAH patients for survival analysis; (b) for diagnosis of CTD, SSc was diagnosed according to the 1980 American College of Rheumatology (ACR) criteria [16], SLE was diagnosed according to the American Rheumatism Association (ARA) criteria [17,18] or the 2012 Systemic Lupus International Collaborating Clinics (SLICC) classification criteria [19], pSS was diagnosed according to the revised criteria proposed by the American European Consensus Group in 2002 [20], and MCTD was diagnosed according to criteria proposed by Sharp [21] or Alarcon-Segovia [22] or Kasukawa [23]; (c) for diagnosis of PAH, patients who underwent right heart catheterization (RHC) assessment were defined by mPAP greater than 25 mmHg at rest, PAWP less than 15 mmHg, and PVR over 3 WU in the absence of other causes of precapillary pulmonary hypertension [2]; (d) results of anti-U1 RNP antibodies were available; (e) all patients were adults over 18 years old; (f) published in English or Chinese.

Studies with the following characters were disqualified: (a) review, conference abstract, letter to editor, case report, or case series less than ten cases; (b) participants were from the same group of patients; (c) data could not be extracted or transformed into usable form.

### 2.3. Study Selection and Quality Assessment

The selection and assessment of studies was performed independently by two investigators (W.X. and R.D.), and the disagreement between two investigators would be resolved by discussion or by a third (Z.M.) investigator’s judgement if no consensus was reached. According to the above inclusion and exclusion criteria, investigators reviewed titles and abstracts for screening, and then carefully read full texts to determine which studies were finally included. Based on the type of research involved, different methods were applied for risk of bias assessment. Cohort studies and case-control studies were evaluated using the Newcastle–Ottawa (NOS) scale (available at: http://www.ohri.ca/programs/clinical_epidemiology/oxford.asp accessed on 15 September 2022); cross-sectional studies were evaluated using Agency for Healthcare Research and Quality (AHRQ) indicators (available at: https://www.ncbi.nlm.nih.gov/books/NBK35156/ accessed on 15 September 2022).

### 2.4. Data Extraction and Data Items

Data were collected independently by two investigators (W.X. and R.D.) using a form and checked after completion. When agreement could not be reached after discussion between two investigators, a third investigator (M.L.) would make the final decision. The data extraction form contained following items: basic information (author, published time, country, study design, sample size), baseline characteristics (CTD type, age, sex, disease duration), and outcomes (number of anti-U1 RNP antibody-positive patients among CTD patients with and without PAH, respectively, or among survivors and non-survivors with CTD-PAH, respectively).

### 2.5. Statistical Analysis

Statistical analysis was performed by Stata15.0 software. Odds ratio (OR) with a 95% confidence interval (CI) was adopted as a measure of effect in risk factor analysis, and hazard ratio (HR) with a 95% confidence interval (CI) was adopted in prognostic factor analysis. A *p* value less than 0.05 was defined to be significant. Heterogeneity between the included studies were evaluated by Q statistic and I^2^ statistic; if I^2^ equaled or was larger than 50% or *p* less than 0.1, heterogeneity would be considered to exist. Outcomes were synthesized using a random effects model when significant heterogeneity was detected; otherwise, a fixed effects model would be selected. Sensitivity analysis and subgroup analysis would be applied for exploring potential sources of heterogeneity. The Egger’s test was conducted for publication bias assessment; a *p* value less than 0.05 was considered to be significant.

## 3. Results

### 3.1. Study Selection

**Figure 1:**
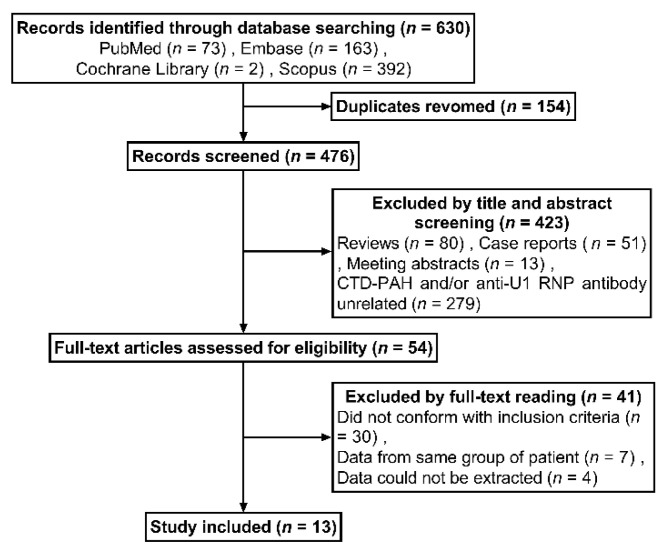
Flow diagram demonstrating the process of study selection. CTD-PAH: connective tissue disease-associated pulmonary arterial hypertension; RNP: ribonucleoprotein.

A total of 630 results were identified through database searching. After removing duplicates, titles and abstracts of 476 studies were screened and 423 studies were excluded. The remaining 54 studies were assessed by full-text review for eligibility. Thirteen studies [10,11,12,13,14,24,25,26,27,28,29,30,31] were ultimately included for analysis. The process of study selection is demonstrated by a flow diagram in Figure 1.

### 3.2. Study Characteristics and Quality Evaluation 

A total of 6671 patients in 13 [10,11,12,13,14,24,25,26,27,28,29,30,31] studies were included in our analysis. The majority of patients were female, and most studies were conducted in Asia. Three studies were concerned with SSc [24,25,26], five with SLE [10,12,13,27,28], two with pSS [29,30], one with myositis [31], and two with multiple types of CTD [11,14]. Basic characteristics and quality evaluation outcomes of these studies are displayed in Table 1. Detailed information and quality evaluation outcomes are available in Tables S2 and S3, respectively. All included studies were of good or medium quality (total score ≥ 6).

### 3.3. Analysis of Relationship between Anti-U1 RNP Antibody and CTD-PAH

#### 3.3.1. Risk Factor Analysis

**Figure 2:**
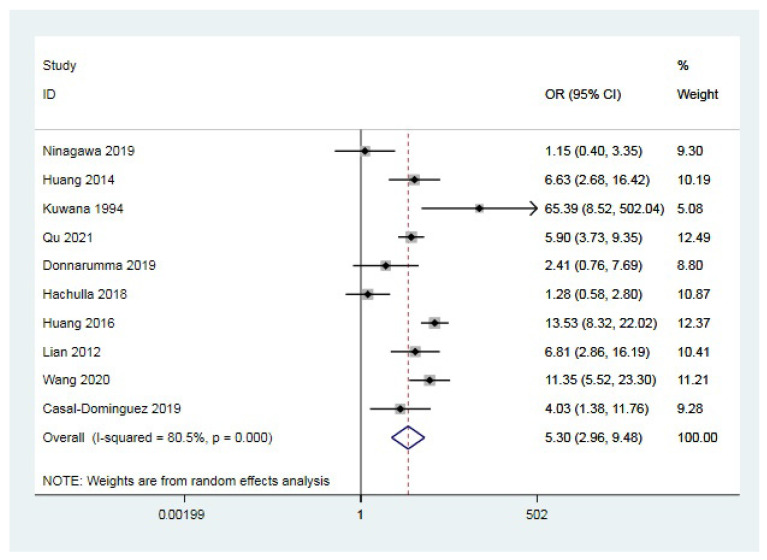
Forest plot of the relationship between anti-U1 RNP antibody and CTD-PAH [10,12,13,24,25,26,27,28,29,31]. OR: odds ratio; CI: confidence interval.

A total of 10 studies [10,12,13,24,25,26,27,28,29,31] contained statistics of the number of anti-U1 RNP antibody-positive patients in the CTD-PAH group and the connective tissue disease without pulmonary arterial hypertension (CTD-no PAH) group, respectively. A random-effect model was adopted as heterogeneity existed (I^2^ =80.5%, *p <* 0.001). The pooled OR was 5.30 (95%CI 2.96–9.48, *p* < 0.001), which indicated anti-U1 RNP antibody positivity as a risk factor for CTD-PAH; the result is shown in Figure 2. In order to determine the source of heterogeneity, a subgroup analysis was conducted based on ethnic background, CTD type, sample size, and diagnostic method. According to the results, ethnic background and sample size may have contributed to the heterogeneity, as shown in Table 2. Studies of Asians showed significant heterogeneity (I^2^ = 75.1%); however, studies of ethnic groups other than Asian (European or American) showed low heterogeneity (I^2^ = 34.2%). In the group of more than one hundred sample members, heterogeneity was high (I^2^ = 78.8%), whereas it was undetectable in the group of equal or less than one hundred members (I^2^ = 0.0%). Figure S1 shows the sensitivity analysis result, which remained stable after removing any included studies. No considerable publication bias was detected based on the result of the Egger’s test (*p* = 0.475).

#### 3.3.2. Prognostic Factor Analysis

**Figure 3:**
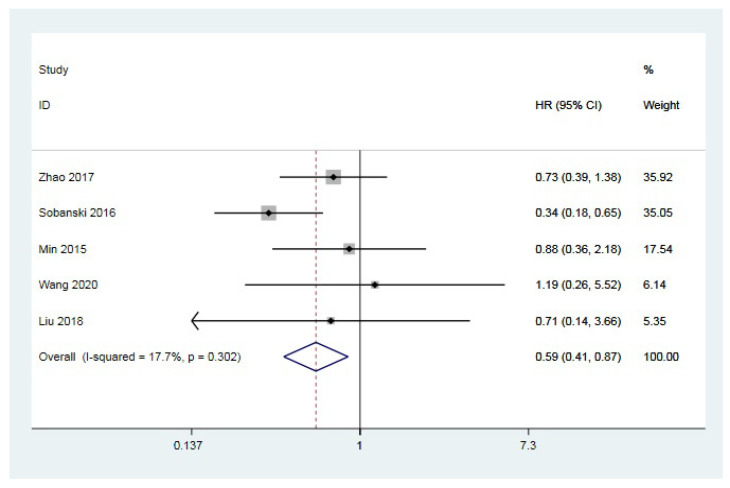
Forest plot of the relationship between anti-U1 RNP antibody positivity and mortality of CTD-PAH. HR: hazard ratio; CI: confidence interval [11,14,29,30].

A total of four studies [11,14,29,30] provided the survival analysis outcomes of anti-U1 RNP-positive and -negative CTD-PAH patients. A fixed-effect model was adopted as there was no considerable heterogeneity (I^2^ =24.7%, *p* = 0.263). The pooled HR was 0.55 (95%CI 0.36–0.83, *p* = 0.005), suggesting that anti-U1 RNP positivity was associated with better survival of CTD-PAH; the result is shown in Figure 3. The publication bias was non-significant according to the result of the Egger’s test (*p* = 0.517).

## 4. Discussion

In patients with CTD, PAH is a severe complication and a major cause of death [32,33]. However, atypical clinical symptoms of CTD-PAH make it hard to achieve prompt recognition. The multiparameter screening approach using a combination of clinical features, NT-proBNP, echocardiography, and pulmonary function tests (PFTs), has been recommended by the latest 2022 ESC/ERS guideline for early detection of PAH [1]. Annual systematic screening is recommended by ESC/ERS guideline in SSc patients due to a relatively high prevalence of PAH (5–19%) [1], but no consensus exists regarding whether echocardiography is necessary in other CTDs, such as SLE, since the prevalence of PAH varies greatly between studies (0.5–17.5%) [34]. During clinical practice, we found that a considerable amount of patients could not insist on taking screening tests annually. Qu et al. established a risk stratification model combining clinical variables and routine autoantibodies, with anti-RNP antibody included, and suggested annual screening tests in SLE patients of high PAH risk [10]. Compared with echocardiography and PFTs, antibody detection is more economical and convenient, and the frequency can be increased to every three or six months for earlier diagnosis. CTD patients at high risk of PAH should be identified and screened systematically; therefore, it makes sense to find an easily detected predictor for the risk of PAH in CTD patients.

Anti-U1 RNP antibody is shared by various CTDs, presenting in 6–17% of SSc patients [35], 13–30% of SLE patients [36], 2–20% of pSS patients [30,37], and 100% of MCTD patients [21]. Considering that anti-U1 RNP antibody is routinely tested in CTD patients and has been reported to correlate with PAH, it is a potentially ideal predictor. Therefore, in order to determine whether anti-U1 RNP antibodies contribute to CTD-PAH, this meta-analysis was performed.

According to the result of this meta-analysis, anti-U1 RNP antibody is a risk factor for PAH among CTD patients with a pooled OR of 5.30 (95%CI 2.96–9.48, *p* < 0.001). A previous meta-analysis about SLE-associated pulmonary arterial hypertension (SLE-PAH) conducted by Wang et al. showed a similar finding [38]. Despite the fact that the exact role of the anti-U1 RNP antibody in CTD-PAH pathogenesis remains unclear, it is predicted that this antibody may contribute to the PAH development by participating in vasculopathy. An in vitro study suggested that the anti-U1 RNP antibody extracted from CTD patients could bind with human pulmonary arterial endothelial cell (HPAEC) and directly recognize a variety of antigens on its surface, being a possible trigger of endothelial cell inflammation of CTD-PAH [39]. Furthermore, anti-U1RNP antibody has been shown to up-regulate the expression of intercellular adhesion molecule-1, endothelial leucocyte adhesion molecule-1, and class II major histocompatibility complex molecules in human HPAECs [40].

Distinct heterogeneity presented in the analysis of risk factors; one of the possible sources was ethnic background, revealed by subgroup analysis. The impact of racial factors on PAH has already been illustrated by former research. Studies have already demonstrated the impact of racial factors on PAH. According to SSc patients, Asians have a higher prevalence of PAH than white people, regardless of their geographical location. Asian patients were reported to have a higher prevalence of PAH compared with white patients, independent of geographical location, in SSc patients [41]. Another study reported that African Americans were more likely to develop PAH than Caucasians [42]. The ethnic difference in the frequency of the anti-U1 RNP antibody may play a role as well. For example, Asian SSc patients were reported to have both higher positive rates of anti-U1-RNP antibodies and higher mortality than white patients [43]. Furthermore, anti-U1-RNP antibodies were found to be most prevalent in Afro-Caribbeans among a group of SLE patients consisting of Europeans, Afro-Caribbeans, and Asians [44].

The anti-U1 RNP antibody appears to be protective against mortality in CTD-PAH, with a pooled HR of 0.57 (95% CI 0.38–0.85, *p* = 0.006). A study conducted by Sobanski et al. compared the clinical characteristics of CTD-PAH patients with and without anti-U1 RNP antibodies: anti-U1 RNP-positive patients had younger ages, shorter CTD duration, and milder functional impairments (lower WHO functional class, longer 6-min walk distance, higher diffusion capacity for carbon monoxide) when PAH was diagnosed. In contrast, hemodynamic parameters, including mPAP and PVR, were similar to those of anti-U1 RNP-negative participants [11]. Anti-U1 RNP-positive groups have a higher proportion of SLE patients, which may explain the better survival, since SSc patients have a relatively poor prognosis. Different histopathological characteristics may contribute to their different prognosis. Unlike SLE-PAH, which is characterized by plexogenic arteriopathy and fibrinoid vasculitis, SSc-associated pulmonary arterial hypertension (SSc-PAH) is typically characterized by fibrous intimal thickening of medium-sized arteries and branching small vessels. Luminal occlusion of medium to small arteries can also induce impairment of gas exchange, leading to respiratory failure [45]. As a result, conventional therapy including immunosuppressive agents and pulmonary vasodilators are effective in SLE-PAH patients, while SSc-PAH patients are unable to respond to immunosuppressive therapy, and, so far, SSc-PAH has not been treated satisfactorily [46]. The data available in studies involved in the survival analysis were inadequate for subgroup analysis of different CTD types. Qian et al. made a comment on Sobanski’s research and raised questions about the potential negative impact of RNP status on survival of SLE-PAH [47]. Hence, we conducted an additional analysis by combining the existing statistics with new statistics provided by Qian et al. and Sobanski et al. in reply. The result showed that anti-U1 RNP positivity was associated with better survival of SSc-PAH with a pooled HR of 0.47 (95%CI 0.26–0.85, *p* = 0.013), while its association with survival of SLE-PAH was not significant with a pooled HR of 1.69 (95%CI 0.77–3.70, *p* = 0.189). The details are shown in Table S4. However, anti-U1 RNP positivity was associated with better survival regardless of sex, age, functional, or hemodynamic parameters in Sobanski’s research [11]. Therefore, there is a hypothesis that a serologic homogeneity is carried by the anti-U1 RNP antibody among CTD-PAH patients, though the underlying mechanism was uncertain.

Several limitations exist in this meta-analysis. Firstly, since the vast majority of the population involved in this meta-analysis were Asians, the results may not be applicable to all patients with other ethnic backgrounds. Furthermore, among all studies included in prognostic factor analysis, the study of Sobanski et al. was the only one showing a significant association between anti-U1 RNP positivity and better survival; the results of other studies were not statistically significant. Therefore, the analytical result was not totally robust. To gain a stronger conclusion, further insights into a larger cohort is required.

## 5. Conclusions

This meta-analysis identified the anti-U1 RNP antibody as a risk factor for PAH in CTD patients. Thus, to diagnose and treat PAH early, regular screening tests such as echocardiography are necessary for patients with anti-U1 RNP-positive CTDs. Anti-U1 RNP positivity was also proved to be associated with better survival in CTD-PAH. Further insight into the pathogenic role of the anti-U1-RNP antibody is needed, since anti-U1 RNP-positive patients may belong to a unique subset of CTD with a distinct phenotype.

## Figures and Tables

**Table 1:** Basic characteristics and quality evaluation outcomes of included studies. CTD: connective tissue disease; SSc: systemic sclerosis; SLE: systemic lupus erythematosus; pSS: primary Sjogren’s syndrome; PAH: pulmonary arterial hypertension; RHC: right heart catheterization; NA: not available.

Study	Country	Study Type	CTD Type	Population ^a^	Quality Assessment Score ^b^
Zhao 2017 [14]	China	Cohort	SSc,SLE,pSS	149/41	8
Sobanski 2016 [11]	UK	Cohort	SSc,SLE,pSS	92/250	8
Ninagawa 2019 [24]	Japan	Cross-section	SSc	24/34	6
Huang 2014 [25]	China	Case-control	SSc	25/141	8
Kuwana 1994 [26]	Japan	Cohort	SSc	19/227	8
Qu 2021 [10]	China	Cohort	SLE	92/3532	8
Donnarumma 2019 [13]	Brazil	Case-control	SLE	21/44	8
Hachulla 2018 [12]	France	Case-control	SLE	51/101	7
Huang 2016 [27]	China	Case-control	SLE	111/444	8
Lian 2012 [28]	China	Case-control	SLE	41/106	8
Wang 2020 [29]	China	Case-control	pSS	103/526	6
Liu 2018 [30]	China	Cohort	pSS	22/7	8
Casal-Dominguez 2019 [31]	US	Cohort	Myositis	39/426	7

^a^ The statistics are presented in the form of: “CTD-PAH/CTD-nPAH” or “Survivors/Non-Survivors”. ^b^ Cohort studies and case-control studies were evaluated using the NOS scale, cross-sectional studies were evaluated using AHRQ indicators.

**Table 2:** Results of subgroup analysis of risk factor analysis. CTD: connective tissue disease; SLE: systemic lupus erythematosus; SSc: systemic sclerosis; OR: odds ratio; CI: confidence interval.

Subgroup	Number of Studies	Pooled OR	95%CI	Heterogeneity	
				I^2^	*p*
Ethnic background					
Asian	7	7.46	4.16–13.38	75.1%	<0.001
Others	3	2.13	1.05–4.30	34.2%	0.219
CTD type					
SLE	5	4.67	2.08–10.45	85.8%	<0.001
SSc	3	6.66	1.00–44.49	85.8%	0.001
Others	2	7.32	2.68–19.97	59.7%	0.115
Sample size					
>100	8	6.80	3.77–12.26	78.8%	<0.001
≦100	2	1.62	0.74–3.55	0.0%	0.359

## Data Availability

Not applicable.

## Supplementary Materials

The following supporting information can be downloaded at: https://www.mdpi.com/article/10.3390/jcm12010013/s1, Table S1: Searching strategy in different databases; Table S2: Characteristics of included studies; Table S3: Quality evaluation outcomes; Table S4: Results of subgroup analysis of prognostic factor analysis; Figure S1: Sensitivity analysis of risk factor analysis.
