# Supplementary material for: The Role of Anti-U1 RNP Antibody in Connective Tissue Disease-Associated Pulmonary Arterial Hypertension: A Systematic Review and Meta-Analysis
Source: J Clin Med. 2022 Dec 20;12(1):13. doi: 10.3390/jcm12010013 (PMC9821587; doi:10.3390/jcm12010013)
Supplement: Supplementary file 1 [file jcm-12-00013-s001.zip › supplementary table S1.pdf]

Table S1. Searching strategy in different databases.

Database	Searching Strategy
PubMed/ Scopus	1# (Pulmonary arterial hypertension) OR (Pulmonary hypertension) OR (PAH)<br>2# (Anti-U1 RNP antibody) OR (Anti-nRNP antibody) OR (Anti-RNP antibody) OR (Anti-ribonucleoprotein antibody)<br>3# 1# AND 2#
Embase	1# 'Pulmonary arterial hypertension':ab,ti OR 'Pulmonary hypertension':ab,ti OR 'PAH':ab,ti<br>2# 'Anti-U1 RNP antibody':ab,ti OR 'Anti-nRNP antibody':ab,ti OR 'Anti-RNP antibody':ab,ti OR 'Anti-ribonucleoprotein antibody':ab,ti<br>3# 1# AND 2#
Cochrane Library	1# (Pulmonary arterial hypertension):ti,ab,kw OR (Pulmonary hypertension):ti,ab,kw OR (PAH):ti,ab,kw<br>2# (Anti-U1 RNP antibody):ti,ab,kw OR (Anti-nRNP antibody):ti,ab,kw OR (Anti-RNP antibody):ti,ab,kw OR (Anti-ribonucleoprotein antibody):ti,ab,kw<br>3# 1# AND 2#
