# Supplementary material for: The Role of Anti-U1 RNP Antibody in Connective Tissue Disease-Associated Pulmonary Arterial Hypertension: A Systematic Review and Meta-Analysis
Source: J Clin Med. 2022 Dec 20;12(1):13. doi: 10.3390/jcm12010013 (PMC9821587; doi:10.3390/jcm12010013)
Supplement: Supplementary file 1 [file jcm-12-00013-s001.zip › supplementary table S2.pdf]

Table S2. Characteristics of included studies. CTD: Connective tissue disease; SSc: Systemic sclerosis; SLE: Systemic lupus erythematosus; pSS: primary Sjogren's syndrome; PAH: pulmonary arterial hypertension; CTD-PAH: connective tissue disease-associated pulmonary arterial hypertension; CTD-no PAH: connective tissue disease without pulmonary arterial hypertension; RHC: Right heart catheterization; mPAP: mean pulmonary arterial pressure; NA: Not available.

Table S2-1. Characteristics of studies included in risk factor analysis.

Study	Country	Study type	CTD type	Population[a]	Female (%)[a]	Age (years)[a,b]	Disease duration (years)[a,b]	mPAP of patients with PAH (mmHg)[b]	Anti-U1RNP -positive population[a]
Ninagawa 2019 [24]	Japan	Cross-section	SSc	24/34	87.5%/88.2%	58.0±5.3/63.7±6.1	NA	NA	10/13
Huang 2014 [25]	China	Case-control	SSc	25/141	92.0%/91.5%	44.8±10.8/44.7±12.5	7.6±7.3/6.2±7.1	43.3±11.8	15/26
Kuwana 1994 [26]	Japan	Cohort	SSc	19/227	88.40%	NA	3.6±3.0	NA	18/49
Qu 2021 [10]	China	Cohort	SLE	92/3532	98.9%/93.9%	25.0±7.5/27.6±11.0	0.42±0.86/0.17±0.34	NA	66/1062
Donnarumma 2019 [13]	Brazil	Case-control	SLE	21/44	95.2%/95.5%	NA	6.7±6.4/5.6±3.2	48.9±11.7	11/19
Hachulla 2018 [12]	France	Case-control	SLE	51/101	91.8%/92.1%	47.6±12.2/46.9±16.2	NA	47.6±12.2	14/37
Huang 2016 [27]	China	Case-control	SLE	111/444	97.3%/97.3%	34.6±8.6/34.6±8.5	7.0±6.3/3.9±4.6	46.4±11.4	70/52
Lian 2012 [28]	China	Case-control	SLE	41/106	85.4%/86.8%	26.5±11.5/29.7±13.6	NA	NA	33/40
Wang 2020 [29]	China	Case-control	pSS	103/526	98.1%/96.4%	37.4±13.1/44.1±13.1	4.5±5.5/3.4±3.8	48.1±10.7	23/13
Casal-Dominguez 2019 [31]	US	Cohort	Myositis	39/426	75.5%	NA	NA	NA	5/15

[a] The statistics are presented in the form of “CTD-PAH/ CTD-no PAH”. [b] The statistics are presented in the form of “mean ± SD”.

Table S2-1. Characteristics of studies included in prognostic factor analysis.

Study	Country	Study type	CTD type	Population[a]	Female (%)[a]	Age (years)[a,b]	Disease duration (years)[a,b]	mPAP of patients with PAH (mmHg)[b]
Zhao 2017 [14]	China	Cohort	SSc,SLE,pSS	149/41	96.0%/95.1%	36.9±10.3/41.3±9.8	6.5±6.1/9.0±8.1	46.2±10.4
Sobanski 2016 [11]	UK	Cohort	SSc,SLE,pSS	92/250	85.4%	60.2±13.2	NA	40.3±12.6
Wang 2020 [29]	China	Case-control	pSS	103/526	98.1%/96.4%	37.4±13.1/44.1±13.1	4.5±5.5/3.4±3.8	48.1±10.7
Liu 2018 [30]	China	Cohort	pSS	22/7	100%/100%	40.4±10.0/41.3±5.4	4.9±5.1/10.2±7.5	51.8±10.0

[a] The statistics are presented in the form of “Survivors/Non-Survivors”. [b] The statistics are presented in the form of “mean ± SD”.
