# Supplementary material for: The Role of Anti-U1 RNP Antibody in Connective Tissue Disease-Associated Pulmonary Arterial Hypertension: A Systematic Review and Meta-Analysis
Source: J Clin Med. 2022 Dec 20;12(1):13. doi: 10.3390/jcm12010013 (PMC9821587; doi:10.3390/jcm12010013)
Supplement: Supplementary file 1 [file jcm-12-00013-s001.zip › supplementary table S3.pdf]

**Table S3.** Quality evaluation outcomes.

**Table S3-1.** NOS quality evaluation case-control studies.

| **Items** | **Huang 2014** [28] | **Donnarumma 2019** [13] | **Hachulla 2018** [12] | **Huang 2016** [27] | **Lian 2012** [28] | **Wang 2020** [29] |
|---|---|---|---|---|---|---|
| Selection | | | | | | |
| Adequate case definition | 1 | 1 | 1 | 1 | 1 | 1 |
| Representativeness of the cases | 1 | 1 | 1 | 1 | 1 | 1 |
| Selection of controls | 0 | 0 | 0 | 0 | 0 | 0 |
| Definition of controls | 1 | 1 | 1 | 1 | 1 | 1 |
| Comparability | 2 | 2 | 2 | 2 | 2 | 0 |
| Exposure | | | | | | |
| Ascertainment of exposure | 1 | 1 | 1 | 1 | 1 | 1 |
| Same method of ascertainment for cases and controls | 1 | 1 | 1 | 1 | 1 | 1 |
| Non-response rate | 1 | 1 | 0 | 1 | 1 | 1 |
| **Total score** | 8 | 8 | 7 | 8 | 8 | 6 |

**Table S3-2.** NOS quality evaluation for case-sectional studies for cohort studies.

| Items | Zhao **2017** [14] | Sobanski **2016** [11] | Kuwana **1994** [26] | Qu **2021** [10] | Liu **2018** [30] | Casal-Dominguez **2019** [31] |
|---|---|---|---|---|---|---|
| Selection | | | | | | |
| Representativeness of the exposed cohort | 1 | 1 | 1 | 1 | 1 | 1 |
| Selection of the non exposed cohort | 1 | 1 | 1 | 1 | 1 | 1 |
| Ascertainment of exposure | 1 | 1 | 1 | 1 | 1 | 1 |
| Demonstration that outcome of interest was not present at start of study | 1 | 1 | 1 | 1 | 1 | 0 |
| Comparability | 1 | 1 | 1 | 1 | 1 | 1 |
| Outcome | | | | | | |
| Ascertainment of outcome | 1 | 1 | 1 | 1 | 1 | 1 |
| Follow-up long enough for outcomes to occur | 1 | 1 | 1 | 1 | 1 | 1 |
| Adequacy of follow up of cohorts | 1 | 1 | 1 | 1 | 1 | 1 |
| **Total score** | 8 | 8 | 8 | 8 | 8 | 7 |

**Table S3-3.** AHRQ indicators quality evaluation for case-sectional studies.

| **Items** | **Ninagawa 2019** [24] |
| --- | --- |
| Define the source of information (survey, record review) | Y |
| List inclusion and exclusion criteria for exposed and unexposed subjects (cases and controls) or refer to previous publications | Y |
| Indicate time period used for identifying patients | Y |
| Indicate whether or not subjects were consecutive if not population-based | Y |
| Indicate if evaluators of subjective components of study were masked to other aspects of the status of the participants | Y |
| Describe any assessments undertaken for quality assurance purposes (e.g. test/retest of primary outcome measurements) | U |
| Explain any patient exclusions from analysis | Y |
| Describe how confounding was assessed and/or controlled | N |
| If applicable, explain how missing data were handled in the analysis | N |
| Summarize patient response rates and completeness of data collection | N |
| Clarify what follow-up, if any, was expected and the percentage of patients for which incomplete data or follow-up was obtained | N |
| **Total score(Y=1, N/U=0)** | 6 |
