# Supplementary material for: The Role of Anti-U1 RNP Antibody in Connective Tissue Disease-Associated Pulmonary Arterial Hypertension: A Systematic Review and Meta-Analysis
Source: J Clin Med. 2022 Dec 20;12(1):13. doi: 10.3390/jcm12010013 (PMC9821587; doi:10.3390/jcm12010013)
Supplement: Supplementary file 1 [file jcm-12-00013-s001.zip › supplementary table S4.pdf]

Table S4. Results of subgroup analysis of prognostic factor analysis. CTD: Connective tissue disease; SSc: Systemic sclerosis; SLE: Systemic lupus erythematosus; HR: Hazard ratio; CI: Confidence interval.

CTD type	Study	HR	95%CI	Weight (%)	*p* value
SSc	Qian 2016 [47]	0.48	(0.19,1.20)	41.1	
	Sobanski 2016 [11]	0.27	(0.22, 1.01)	58.9	
	**Pooled HR**	0.47	(0.26, 0.85)	100.0	0.013
SLE	Qian 2016 [47]	1.46	(0.63, 3.38)	88.1	
	Sobanski 2016 [11]	5.10	(0.52, 50.0)	11.9	
	**Pooled HR**	1.69	(0.77, 3.72)	100.0	0.189
