# Supplementary figures and images for: The Role of Anti-U1 RNP Antibody in Connective Tissue Disease-Associated Pulmonary Arterial Hypertension: A Systematic Review and Meta-Analysis
Source: J Clin Med. 2022 Dec 20;12(1):13. doi: 10.3390/jcm12010013 (PMC9821587; doi:10.3390/jcm12010013)

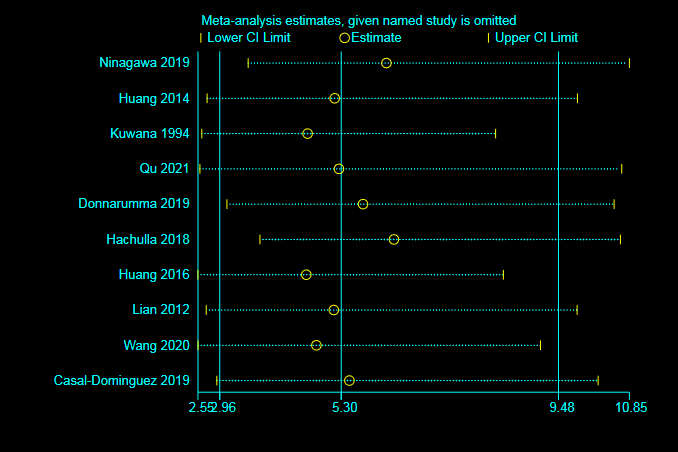

Supplement: Supplementary file 1 [file jcm-12-00013-s001.zip › supplementary figure S1.tif]
